# Surveillance Outcomes in an Australian Cohort Undergoing Piecemeal Polypectomy for Conventional Adenomas and Serrated Lesions

**DOI:** 10.1007/s10620-026-10024-6

**Published:** 2026-06-04

**Authors:** Shweta Sharma, Patricia C. Valery, Katherine Hanigan, Kimberley Ryan, Mark Appleyard, Nicholas Tutticci, Timothy O’Sullivan, Barbara Leggett

**Affiliations:** 1 School of Medicine, University of Queensland, St Lucia, QLD Australia; 2 Department of Gastroenterology, Surgical, Treatment and Rehabilitation Service (STARS), Herston, QLD Australia; 3 QIMR Berghofer, Herston, QLD Australia; 4 Department of Gastroenterology, Royal Brisbane and Women’s Hospital, Herston, QLD Australia; 5 School of Nursing, Faculty of Health, Queensland University of Technology, Herston, QLD Australia; 6 Department of Gastroenterology, Queen Elizabeth II Jubilee Hospital (QEII), Coopers Plains, QLD Australia

**Keywords:** Piecemeal, Surveillance, Recurrence, Cold snare polypectomy

## Abstract

**Background and Aims:**

Current guidelines recommend early surveillance following piecemeal polypectomy due to recurrence risk; however, this contributes substantially to colonoscopy burden. We aimed to identify subgroups in which surveillance can be safely deferred beyond current guideline intervals**.**

**Methods:**

We performed a two-center retrospective study of consecutive patients undergoing first (SC1) and second (SC2) surveillance colonoscopy following piecemeal resection. Primary outcome was histological recurrence; secondary outcome was detection of additional high-risk polyps.

**Results:**

A total of 221 lesions, 130 conventional adenomas (CA) and 91 serrated lesions (SL), were resected piecemeal in 202 patients, with 76% removed using cold snare technique. Recurrence following cold resection occurred in 11% of CA ≥ 20 mm and 15% of CA < 20 mm, while recurrence in SL was low (5% for < 20 mm; 0% for ≥ 20 mm). Hot snare resection was predominantly used for larger CA (79% ≥ 20 mm) and was associated with a recurrence rate of 14%. At SC1, additional high-risk polyps were detected in 27% of patients, particularly following resection of larger index lesions. No advanced neoplasia was detected at SC2 in patients with serrated lesions without recurrence at SC1.

**Conclusions:**

In this real-world study, most piecemeal polypectomy was performed using cold snare technique. There was a clinically significant risk of recurrence in CA including those < 20 mm supporting current recommendations of surveillance at 6 months unless technical advances can be shown to reduce recurrence. Our findings suggest a lower risk of recurrence in SL of all sizes and if confirmed on further studies, surveillance intervals may be able to safely lengthen in these cases.

**Supplementary Information:**

The online version contains supplementary material available at https://doi.org/10.1007/s10620-026-10024-6.

## Introduction

Colorectal cancer (CRC) is a result of the stepwise malignant degeneration of precursor lesions. Timely and complete resection of these is an effective way to reduce CRC mortality. However, colonoscopy is invasive and resource intensive; thus, efforts should be directed to those most likely to benefit. Piecemeal polypectomy is becoming a greater part of clinical practice for various reasons. These include mounting support for the efficacy and safety of piecemeal polypectomy, enhanced recognition of sessile serrated lesions, as well as emphasis on colonoscopy quality with efforts to capture wider margins of normal mucosa and avoiding unintentional partial resection [1–4].

Much of the literature guiding surveillance strategy following piecemeal resection relies on outcomes for larger, i.e.,  ≥ 20 mm, conventional adenomas managed in expert centers. Factors such as operator technique and nature of the lesion, i.e., size, histology, morphology, and location are significant variables. Broadly, however, compared to en bloc resection, recurrence with piecemeal polypectomy is significantly higher and has been quoted up to 20% versus about 3% for en bloc resection [5]. Current piecemeal surveillance strategies are conservative and place a heavy colonoscopy burden on patients and health services.

Current Australian guidelines are consistent with most international recommendations of first surveillance colonoscopy (SC1) after piecemeal polypectomy of a polyp ≥ 20 mm at 6 months followed by a second surveillance colonoscopy (SC2) at 12–18 months [6–11]. Although most guidelines discuss that sessile serrated histology and absence of high-grade dysplasia in the resected specimen are likely to reduce the risk of recurrence after piecemeal polypectomy, they do not give a specific recommendation based on these factors. Most guidelines do not give a specific recommendation for surveillance after piecemeal polypectomy of polyps < 20 mm except for the recent recommendations of the Alberta Colorectal Cancer Screening Program which recommends surveillance at 6 months after piecemeal polypectomy of polyps ≥ 10 mm [12]. As yet, guidelines do not address whether the piecemeal polypectomy has been performed by hot snare or cold snare technique but are largely based off historical piecemeal hot snare resection data of ≥ 20 mm conventional adenomas managed in expert centers.

Conventional adenomas that are larger than 20 mm in size are more likely to be higher risk lesions, harbor covert cancer, and be associated with synchronous advanced pathology [13]. Sessile serrated lesions without dysplasia on the other hand are at a low risk of malignancy even when the size exceeds 10 mm [14]. The risk for these two pathologies may not be comparable and outcomes for smaller lesions are less clearly defined. Furthermore, this landscape has transformed in the last 5 years with margin thermal ablation has reduced recurrence to < 5% in polyps ≥ 20 mm in expert centers and has called into question whether intensive surveillance is still necessary [15]. This technique is being studied but not currently used post-cold-snare piecemeal polypectomy.

The aim of the present study was to clarify whether extrapolating the evidence for piecemeal polypectomies for lesions ≥ 20 mm in size to those < 20 mm leads to over-surveillance. Outcomes were stratified for conventional adenomas versus sessile serrated lesions. We focused on cold snare polypectomy as it was most common and at the time of this cohort, margin thermal ablation was not universally used after hot snare polypectomy. The primary outcome was the frequency of confirmed histological recurrence. As a secondary outcome, we assessed the prevalence of additional high-risk pre-malignant or cancerous lesions at first surveillance. We hoped to identify subgroups in which surveillance could safely be lengthened.

## Methods

### Study Population

The Royal Brisbane and Women’s Hospital (RBWH) and the Surgical Treatment and Rehabilitation Service (STARS) in Brisbane, Australia, have maintained a prospective database for every patient undergoing colonoscopy from 2018 onwards. The RBWH is a 929-bed quaternary and tertiary referral teaching hospital, and the STARS is a 182-bed tertiary public hospital specializing in rehabilitation, elective surgery and endoscopy. This is a retrospective observational cohort study which included consecutive patients aged ≥ 18. Data included all patients undergoing a complete colonoscopy over two years (2018–2019) with a repeat procedure within 3 years of the index colonoscopy. All patient medical records were reviewed to identify those who underwent a piecemeal polypectomy and to collect data on resection technique at index procedure and scar identification as well as additional polyp findings at surveillance.

Exclusion criteria consisted of any of the polyps having prior attempted resection or if the procedure report specifically highlighted that the interval for follow-up colonoscopy was dictated by a reason other than piecemeal polyp surveillance, i.e., underlying diagnosis of inflammatory bowel disease, known or suspected polyposis syndromes, quality of bowel preparation, recent history of colorectal cancer, or high-risk lesions or concerns about incomplete resection. Lastly, if a patient underwent a repeat colonoscopy sooner than planned due to new onset of symptoms, these were excluded.

The optimal management strategy for ≥ 20 mm polyps resected with hot snare piecemeal endoscopic mucosal resection followed by margin thermal ablation is well defined and recommended by international guidelines [16]. This was not standard practice during the study period and therefore its use was sparse. Hence cases post hot snare polypectomy; with or without margin thermal ablation; were excluded from the initial recurrence analysis. For the purposes of all subsequent analyses, i.e., assessment of additional polyps at surveillance as well as outcomes at SC2, all cases of piecemeal polypectomy were included.

### Ethics

The study was approved by the Metro North Health Human Research Ethics Committee (EC00172).

### Procedure

#### Index Colonoscopy

The procedures were performed by a specialist gastroenterologist or advanced trainee under direct supervision. Anesthetist-directed sedation with a combination of fentanyl, midazolam, and propofol was employed in all cases. High definition colonoscopes (HQ190/EZ1500; Olympus, Tokyo, Japan) and carbon dioxide insufflation were used. Patients received split-dose bowel preparation. This is standard for both centers and did not change during the period of this study.

The solution used for submucosal fluid injection was a combination of succinylated gelatin (Gelofusine; B Braun, Bella Vista, Australia) and methylene blue or indigo carmine with or without the use of dilute adrenaline. Dedicated cold snares including Captivator™ Cold single-use snare. Alternatives included Captivator™ II cold and hot single-use snare (Boston Scientific, Marlborough, Mass, USA) or Snare Master Plus hybrid hot and cold snare (Olympus, Tokyo, Japan). Resection technique, i.e., hot or cold resection, choice of snare, as well as decision to utilize a distal attachment cap, defect closure with clips, or snare-tip soft coagulation to defect margin, along with tattoo to locate the site at surveillance was at the proceduralist’s discretion based on their understanding of the evidence at the time. For hot polypectomy, a microprocessor-controlled electrosurgical generator (Endocut effect 3, VIO 300D; ERBE Elektromedizin, Tübingen, Germany) with fractionated current was used in both centers. Where used, margin thermal ablation was performed using STSC (ERBE VIO 300D, SOFT COAG: 80W, Effect 4; ERBE, Tubingen, Germany).

#### Surveillance Colonoscopy

The timing of first and second surveillance was determined by the proceduralist based on their confidence of complete resection, quality of prep, known residual polyps, as well as patient factors such as overall health and availability.

The method of examination of polypectomy site, i.e., use of narrow-band imaging and magnification as well as decision for biopsy of the scar was proceduralist dependent. Lastly, management of recurrence followed by subsequent surveillance recommendation was also proceduralist determined. In the absence of recurrence, outcomes for second surveillance colonoscopy were reviewed.

#### Data Collection

If the patient had both conventional adenoma and a serrated lesions resected piecemeal at index procedure, only the larger lesion was included in analysis. To capture frequency of confident scar identification, all surveillance reports were manually reviewed for either written documentation or photo-documentation of the scar by a single researcher (SS).

The “right colon” was defined as including the caecum, ascending colon, hepatic flexure, and transverse colon. The “left colon” includes the splenic flexure, sigmoid colon, descending colon, and rectum.

Those who were found to have any additional polyps on surveillance colonoscopies were divided into “low-risk,” “high-risk,” and “very-high-risk” groups. Those who were only found to have additional polyps that could safely wait for 5 years or longer for surveillance colonoscopy as per the Australian guidelines were considered “low risk.” “High-risk” pathology on surveillance colonoscopy was defined as those who would be recommended to undergo a surveillance colonoscopy in 3 years’ time as per the Australian guidelines. Similarly, as per the national guidelines, those who would be recommended to return for surveillance in 1 year were defined as “very high risk” [17]. (Supplementary Table 1).

## Statistical Analysis

Analyses were conducted using StataNow/MP (version 19.5; StataCorp LLC, College Station, TX). Univariable methods were used to describe the cohort and assess differences according lesion size (≥ 20 mm vs < 20 mm). Continuous and normally distributed variables were presented as mean ± SD, and differences between groups were analyzed by one-way ANOVA. Non-normally distributed data are presented as median (range) and were analyzed using the Kruskal–Wallis H test. Categorical data were presented as proportional percent and analyzed using Pearson’s chi-squared (*χ*^2^) or Fisher’s exact test as denoted. Statistical significance was set at alpha = 0.05. Granular outcome analysis for high- versus very-high-risk additional polyps on surveillance could not be performed due to small numbers. Analysis of second surveillance data was no feasible due to rare event rate.

Multivariable logistic regression analysis reported in terms of odds ratios (OR) with associated 95% confidence intervals (CI) was used to examine factors associated with the following: (i) recurrence among 168 polyps and (ii) additional high- or very-high-risk polyps at first surveillance colonoscopy among 202 patients. First, unadjusted ORs are presented. Considering our understanding of the relationships among variables and their clinical relevance, as well as associations assessed first in univariate models and then in a multivariable analysis, we employed forward stepwise selection (*p*-value for addition < 0.20).

## Results

**Fig. 1:**
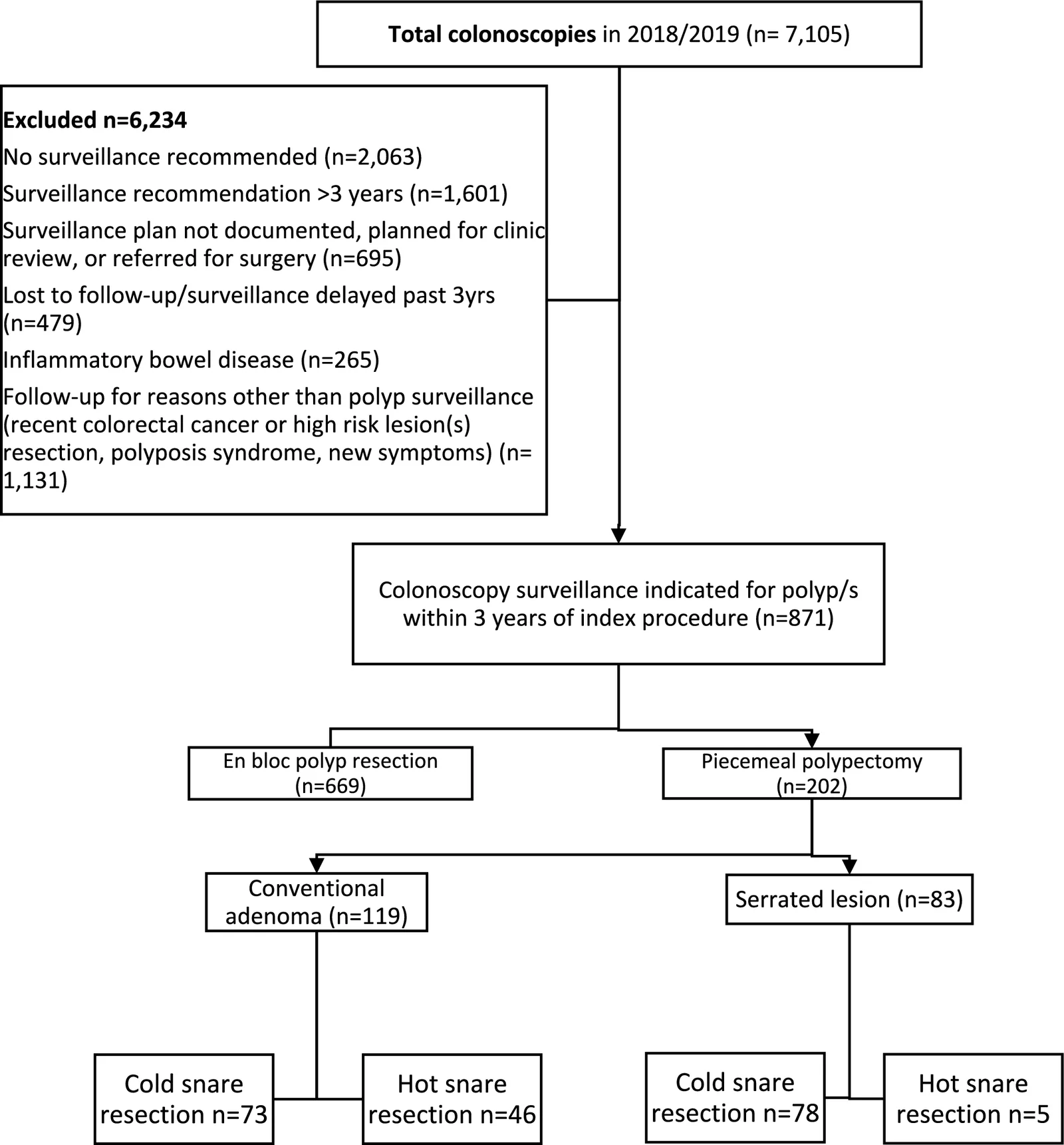
Exclusion criteria and flow of patients through the study.

In the years 2018 and 2019, 7,105 individuals underwent a colonoscopy at the two centers. The mean cecal intubation rate for the whole colonoscopy cohort through the study period was 97.8% with the mean adenoma detection rates of 53.1% and sessile serrated lesion detection rate of 15.5%. In 871 of these procedures, surveillance within 3 years was recommended because of high-risk polyps identified at the index colonoscopy. 669 of these were excluded from analysis due to en bloc resection method. A significant number of 202 (23%) were included for analysis due to the use of a piecemeal polypectomy technique (Fig. 1).

Of the patients with polyps resected piecemeal, 119/202 (59%) had a dominant conventional adenoma and 83 (41%) had serrated lesions predominantly sessile serrated lesions (Supplementary Tables 2 and 3). 52% (105/202) of these had polyps that were < 20 mm in size. Cold snare polypectomy technique was utilized in 61% (73/119) of those with a conventional adenoma and 94% (78/83) with serrated lesions. Of the minority of polyps resected by hot snare piecemeal polypectomy, 42/53 (79%) were conventional adenomas ≥ 20 mm. Histological recurrence at SC1 was 6/42 (15%) which likely reflects a low snare-tip soft coagulation rate of 43%. Subsequent analysis is focused on cases resected by cold snare polypectomy.

### Conventional Adenomas Resected by Cold Snare Polypectomy

The patient group were predominantly male with a mean age of 65.1 years (SD = 13.4). Of the 82 polyps resected cold piecemeal, 57% (47/82) were < 20 mm in size (Table 1). The largest resected lesion measured 50 mm. There was a right colon predominance in polyps less than 20 mm in size.

**Table 1:** Characteristics of conventional adenomas in 73 patients with 82 polyps resected with cold snare

	< 20 mm	≥ 20 mm	
Patient characteristics (%)	*N* = 44 (60)*	*N* = 29 (40)*	*p*-value
Age, mean (SD), years	63.7 (11.1)	67.2 (11.8)	0.19†
Female, *n* (%)	14 (31.8)	13 (44.8)	0.26
Polyp characteristics	*N* = 47	*N* = 35	
Size			
Median (IQR), mm	15 (12–15)	22 (20–30)	
Location, *n* (%)			
Right colon	43 (91)	28 (80)	0.19*
Exact location			0.26*
Rectum	0 (0)	2 (6)	
Sigmoid, descending colon, splenic flexure	4 (9)	5 (14)	
Transverse colon, hepatic flexure	8 (17)	8 (23)	
Ascending colon	18 (38)	13 (37)	
Caecum, IC valve, appendiceal orifice	17 (36)	7 (20)	
Histology			0.11*
Tubular adenoma with low grade dysplasia (LGD)	32 (68)	26 (74)	
Tubular adenoma with high-grade dysplasia (HGD)	5 (11)	1 (3)	
Tubulovillous adenoma with LGD	10 (21)	5 (14)	
Tubulovillous adenoma with HGD	0 (0)	3 (9)	
Polypectomy technique, *n* (%)			
Submucosal injection	30 (64)	28 (80)	0.11
Snare tip soft coagulation (STSC) post-polypectomy	1 (2)	1 (3)	1.00*
Clip placed	4 (9)	3 (9)	0.99
Site tattooed	1 (2)	5 (14)	0.079*
Outcome at S1			
Time from index colonoscopy to first surveillance, median (IQR), months	8.0 (6.0–12.0)	6.0 (4.0–7.0)	**< 0.001**‡
Time to S1 < 6 months	9 (19)	14 (40)	**0.038**
Time to ≥ 6 months	38 (81)	21 (60)	
Confident scar identification	24 (51)	25 (71)	0.063
Suspected macroscopic recurrence	9 (19)	3 (9)	0.18
Biopsy/resection	16 (30)	32 (42)	0.19
Histological recurrence	7 (15)	4 (11)	0.65

Values are *n* (%) and Pearson’s chi-squared are reported unless otherwise defined

*SD* standard deviation, *N* frequency

*Fisher’s exact test; ^†^Two-sample *t* test; ^‡^Wilcoxon rank sum

Bold values indicate statistical significance (*p* < 0.05)

The median time to first surveillance was significantly less in the polyps that were ≥ 20 mm in size with the median time being 6 months (IQR 4.0–7.0) compared to 8 months (IQR 6.0–12.0; *p* < 0.001) for those measuring less than 20 mm. Confident scar identification was 71% in large polyps but only 51% in smaller polyps (*p* = 0.063). Histological recurrence at SC1 was demonstrated in 15% of conventional adenomas < 20 mm. Although this was numerically higher than the 11% rate of histological recurrence in ≥ 20 mm conventional adenomas resected cold piecemeal, this difference was not statistically significant (*p* = 0.65).

### Serrated Lesions Resected by Cold Snare Polypectomy

Patients with serrated lesions were younger than those with conventional adenomas (mean age 55.9 years (SD = 13.7 vs 65.1 years, SD = 11.4; *p* < 0.001) (Table 2). Of the 86 polyps resected with cold snare polypectomy technique, 25 (29%) were ≥ 20 mm. Maximum lesion size was 35 mm with the smallest lesion resected cold piecemeal measuring 6 mm. Higher-risk lesions, i.e., SSL-D and TSAs, were infrequent with a total of 3 and 2 polyps, respectively.

**Table 2:** Characteristics of serrated lesions in 78 patients with 86 polyps resected using cold snare

	< 20 mm	≥ 20 mm	
Patient characteristics (%)	*N* = 55 (71)*	*N* = 23 (29)*	*p*-value
Age, mean (SD), years	55.7 (14.3)	56.5 (12.4)	0.83†
Sex, female, *n* (%)	40 (72.7)	16 (69.6)	0.78
Polyp characteristics	*N* = 61	*N* = 25	
Size			
Median (IQR), mm	14 (10–15)	20 (20–25)	
Location, *n* (%)			
Right colon	51 (84)	25 (100)	0.32*
Exact location			0.025*
Rectum	1 (2)	0 (0)	
Sigmoid, descending colon, splenic flexure	9 (15)	0 (0)	
Transverse colon, hepatic flexure	14 (23)	5 (20)	
Ascending colon	21 (34)	17 (68)	
Caecum, IC valve, appendiceal orifice	16 (26)	3 (12)	
Histology, *n* (%)			0.26*
Hyperplastic polyp	8 (13)	1 (4)	
Sessile serrated lesion	50 (82)	22 (88)	
Sessile serrated lesion with dysplasia	1 (2)	2 (8)	
Traditional serrated lesion	2 (3)	0 (0)	
Polypectomy technique			
Submucosal injection	47 (77)	19 (76)	0.92
Snare tip soft coagulation post-polypectomy	1 (2)	0(0)	1.00*
Clip(s) placed	5 (8)	1 (4)	0.67
Site tattooed	2 (3)	0 (0)	1.00*
Outcome at S1			
Time from index procedure to first surveillance, median (IQR), months	9.0 (6.0–13.0)	6.0 (5.0–8.0)	**< 0.001**‡
Time to S1 < 6 months	4 (7)	7 (28)	**0.012**
Time to S1 ≥ 6 months	57 (93)	18 (72)	
Confident scar identification	18 (30)	17 (57)	**0.012**
Suspected macroscopic recurrence	4 (7)	2 (7)	1.00
Biopsy/resection	7 (11)	8 (27)	0.066
Histological recurrence	3 (5)	0 (0)	0.55

Values are *n* (%) and Pearson’s chi-squared are reported unless otherwise defined

*SD* standard deviation, *N* frequency

*Fisher’s exact test; ^†^Two-sample t test; ^‡^Wilcoxon rank sum

Bold values indicate statistical significance (*p* < 0.05)

Time to first surveillance was significantly greater in patients with serrated polyps that were less than 20 mm in size with a median of 9 months (IQR 6.0–13.0) versus 6 months (IQR 5.0–8.0; *p* < 0.001) in polyps greater than 20 mm. Confident scar identification was less common than when conventional adenomas had been resected piecemeal and scar was identified at SC1 in only 30% (18/61) of cases with a serrated polyp < 20 mm resected piecemeal by cold snare polypectomy vs 57% (17/25 polyp ≥ 20 mm (*p* = 0.012). Histological recurrence at SC1 was not common after piecemeal polypectomy of serrated lesions (3.4%, 3/86 polyps), with difference according to lesion size (3/61 in polyps < 20 mm vs none in ≥ 20 mm; *p* = 1.00). Of these 3 cases, one in the ascending colon may have been related to failure to use submucosal injection to further demarcate the lesion borders and two are likely to be associated with their technically difficult location in the appendiceal orifice which is well known to be a challenging location with high risk of recurrence.

### Factors Associated with Histological Recurrence at SC1 After Cold Snare Polypectomy

The combined polyp recurrence rate at first surveillance was 8.3% and was more common in conventional adenomas (Table 3). Those with polyp recurrence were 11.1 years older (mean 71.0 years [SD = 9.9] vs 59.9 years [SD = 13.2]; *p* = 0.003). On univariate analysis, recurrence in the conventional adenoma group was 4.3-fold higher (OR = 4.29, 95% CI 1.15–15.97) as compared to the serrated lesion group (*p* = 0.030) (Table 4). Neither polyp location nor size was statistically significant determinants for recurrence. The only procedural factor significantly associated with recurrence was tattooing. This was associated with an 8.1-fold increased risk of recurrence (95% CI 1.71–38.55; 0.008). Additionally, less recurrence was found in patients brought back for SC1 later (OR = 0.79, 95% CI 0.65–0.98; *p* = 0.03) although this difference disappeared on multivariable analysis (Table 4). Older age was the only factor significantly associated with recurrence (adjusted OR = 2.06, 95% CI 1.09–3.89; *p* = 0.025) on multivariate analysis.

**Table 3:** Factors associated with per-polyp recurrence at first surveillance colonoscopy among 168 polyps resected with cold snare technique

	No recurrence *N* = 154 (91.7%)*	Recurrence *N* = 14 (8.3%)*	*p*-value
Age, mean (SD), years	59.9 (13.3)	71.0 (9.9)	**0.003**
Female, *n* (%)	85 (55.2)	8 (57.1)	0.89
Polyp histology			
Serrated	83 (53.9)	3 (21.4)	0.025*
Conventional adenoma	71 (46.1)	11 (79.0)	
Polyp location (right vs left)			
Right colon	133 (86.4)	14 (100.0)	0.22*
Left colon	21 (13.6)	0 (0.0)	
Polyp size (mm), median (IQR)	15.0 (12.0–20.0)	15.0 (12.0–20.0)	0.92
Polyp size			
< 20 mm	98 (63.6)	10 (71.4)	0.56
≥ 20 mm	56 (36.4)	4 (28.6)	
Submucosal injection	114 (74.0)	10 (71.4)	0.76*
Snare tip soft coagulation post-polypectomy	3 (1.9)	0 (0)	1.0*
Clip(s) placed	11 (7.1)	2 (14.3)	0.34
Site tattooed	5 (3.2)	3 (21.4)	0.020*
Time from index colonoscopy (months) to S1, median (IQR)	7.0 (6.0–12.0)	6.0 (5.0–6.0)	**0.015**

Values are *n* (%) unless otherwise defined

*SD* standard deviation, *N*, frequency

*Fisher’s exact test

Bold values indicate statistical significance (*p* < 0.05)

**Table 4:** Results from logistic regression analysis assessing factors associated with recurrence among 168 polyps with cold snare technique

	OR (95% CI)	*p*-value	Adjusted OR (95% CI)*	*p*-value
Age in decades	**2.37 (1.31–4.29)**	**0.004**	**2.06 (1.09–3.89)**	**0.025**
Sex (female vs male)	1.08 (0.36–3.27)	0.89	N/S	
Polyp histology (conventional adenoma vs serrated)	**4.29 (1.15–15.97)**	**0.03**	2.85 (0.67–12.16)	0.16
Polyp location (right vs left)	1.00	(omitted)	N/S	
Size (≥ 20 mm vs < 20 mm)	0.70 (0.21–2.34)	0.56	0.29 (0.07–1.28)	0.10
Exact size	1.03 (0.97–1.10)	0.38	N/S	
Submucosal injection	0.88 (0.26–2.95)	0.83	N/S	
Snare tip soft coagulation post-polypectomy	1.00	(omitted)	N/S	
Clip(s) placed	2.17 (0.43–10.92)	0.35	N/S	
Site tattooed	**8.13 (1.71–38.55)**	**0.008**	5.96 (0.96–37.13)	0.06
Time from index colonoscopy (months), median (IQR)	**0.79 (0.65–0.98)**	**0.028**	0.68 (0.17–2.82)	0.60

Bold values indicate statistical significance (*p* < 0.05)

*N/S* not selected

*Multivariable logistic regression model included histology, age, and size (≥ 20 mm vs < 20 mm), procedures clip and tattooing, and time from index colonoscopy

### Additional Polyps at SC1 After Cold or Hot Snare Polypectomy

75 (34%) of the total study population who underwent both cold and hot snare polypectomy did not have any additional polyps at SC1. Low-risk polyps were found in 87 (39%), high-risk polyps were found in 26 (12%), and very-high-risk polyps were found in 33 (15%). A reason for early surveillance is the detection and removal of high- or very-high-risk polyps so factors which may predict their occurrence were sought.

The fifty-four patients (26.6%) who had additional high- or very-high-risk polyps at first surveillance colonoscopy were 4 years older (67.0 years, IQR 57.0–73.0) than those who had low-risk or no additional polyps (64.8 years, SD = 12.6 vs 60.7 years (SD = 13.0; *p* = 0.046) and had a larger polyp size (20.0 mm, IQR 15.0–30.0) vs 18.0 mm, IQR 14.0–25.0), respectively; *p* = 0.022) (Supplementary Table 4). In univariate analysis, patients with conventional adenomas appeared to have more high-risk polyps, but this did not reach statistical significance (*p* = 0.103). Older age (OR 1.30, 95% CI (1.00–1.68) and larger polyp size (OR = 1.03, 95% CI (1.01–1.05) increased the risk of having additional high or very-high-risk polyps at first surveillance colonoscopy. In multivariable analysis including histology, age, and polyp exact size, only polyp size was associated with having additional high- or very-high-risk polyps at first surveillance colonoscopy (adj-OR = 1.03, 95% CI 1.00–1.05; *p* = 0.035).

### SC2 Outcomes Post Hot and Cold Snare Polypectomy

After exclusion of the 21 patients with recurrence at SC1, follow-up outcomes for the remaining 181 who had undergone either cold or hot snare polypectomy were reviewed. Of these, 124 (69%) underwent SC2. 76 of them had piecemeal resection of a dominant conventional adenoma at index procedure and 48 had an initial serrated lesion (Supplementary Table 5). The numbers are too small to allow for formal statistical comparisons by lesion size. Histological recurrence was confirmed in one case of < 20 mm serrated polyp where the scar was biopsied, and no recurrence confirmed at SC1. No recurrence was seen in < 20 mm conventional adenoma subgroup but there were a total of 4 cases of recurrence in those with a ≥ 20 mm conventional adenoma resected piecemeal at index colonoscopy. Scars had been reviewed at SC1 in all four of these cases and biopsies were performed in two cases without histological recurrence at SC1.

No cancers were identified on SC1 and SC2. Polyp recurrence was endoscopically treated in all cases with the polypectomy technique at the endoscopist’s discretion.

## Discussion

Our study conducted in the context of contemporary, high-quality colonoscopy practice demonstrates that surveillance after piecemeal polypectomy contributes significantly to the overall burden of surveillance. The convenience and safety of piecemeal polypectomy especially by cold snare technique need to be balanced with the need for increased follow-up frequency. Resource conservation and mitigating delays in access to colonoscopy by attempting to lengthen follow-up intervals are the topics of great interest [18–20]. Surveillance intervals can only safely be lengthened, however, by either identifying those at lower risk or reducing post-polypectomy recurrence by improving procedural quality, i.e., reducing the incidence of recurrence at polypectomy site as well as identifying residual high-risk polyps at the time of index colonoscopy.

The frequency of incomplete resection and recurrence varies in the literature [5, 18, 21–23]. Additionally, studies that provide comparison of outcomes from polypectomies less and greater than 20 mm in size are sparse. One contributor to recurrence is polypectomy technique. The evidence surrounding the application of cold snare resection of polyps < 20 mm has been steadily increasing [24]. This technique has comparable efficacy to hot snare resection for polyps measuring < 10 mm [6, 16]. For medium-sized polyps measuring 10–19 mm, however, the literature is less clear with variable reported rates of recurrence after cold snare piecemeal polypectomy [1, 4, 25, 26]. It is thought to be least appropriate for the resection of larger adenomas ≥ 20 mm due to significantly higher rates of recurrence with cold polypectomy [23, 27]. Currently, international guidelines recommend consideration of conventional, i.e., diathermy-based EMR for ≥ 20 mm nonpedunculated adenomatous polyps [16]. There is an evolving interest in expanding cold snare polypectomy indications and in the present real-world study, significant numbers of conventional adenomas ≥ 20 mm were resected by cold snare piecemeal polypectomy. These were included to give a complete picture of the burden of surveillance following piecemeal polypectomy and to provide data on which to base future studies. Margin thermal ablation to the defect has been shown to dramatically reduce the incidence of recurrence at surveillance compared to conventional resection technique is now recommended as standard practice after hot snare piecemeal polypectomy [16]. This technique is now a firmly established standard of care. Hence, the subgroup with hot snare polypectomy with or without STSC were not the focus of our analysis.

The primary outcome of this study was the assessment of recurrence rates. In those with cold piecemeal resection, we found no significant difference in recurrence with smaller conventional adenomas compared to large adenomas. This was an unexpected finding, and our results may be explained by proceduralist selection bias, i.e., larger conventional adenomas that are technically more difficult to resect or those with higher risk of recurrence based on optical assessment may have been more likely to be removed using electrocautery. It is worth noting that in the present study, the recurrence rate of conventional adenomas < 20 mm resected by cold snare polypectomy remains clinically significant at 15%. justifying SC1 at 6 months regardless of adenoma size unless improvement in techniques can reduce recurrence.

Apart from use of submucosal injection, overlapping tissue and wide margin resection, few auxiliary techniques have been shown to improve recurrence rates post-cold resection. The technique of cold-forceps avulsion with adjuvant snare-tip soft coagulation (CAST) has been employed in cases with nonlifting large nonpedunculated polyps as well as in treating recurrence at surveillance [28]. This demonstrates the role of thermal ablation as a safe and effective adjunct to reduce recurrence outside of conventional EMR. This raises the question of thermal ablation post-cold polypectomy, but this has not been formally evaluated or validated.

The only procedural technique with a trend toward higher risk of recurrence was tattoo placement. This could be explained by the possibility that proceduralists were more likely to place a tattoo when additional macroscopic predictors of recurrence risk are macroscopically evident or alternatively when their confidence in complete resection was lower [29]. This trend was also noted where risk of recurrence is lower in those who underwent later first surveillance. Given the small numbers, chance cannot be ruled out; however, this may indicate the value of proceduralist confidence in complete resection and risk identification.

Mirroring the trend in the literature for serrated lesions, recurrence rates were very significantly lower in this subgroup as compared to conventional adenomas [23, 30]. Owing to high rates of complete resection, routine use of this method for polyps < 20 mm is accepted. Additionally, our results support the extension of this technique to large sessile polyps ≥ 20 mm [3, 23, 31–33]. Typically, size is thought to be an important factor where larger lesions are more likely to be associated with recurrence. Like conventional adenomas, we found paradoxically the only 3 cases of recurrence in polyps measuring < 20 mm and all 3 of the lesions had specific factors, i.e., lack of utilization of submucosal injection as well as location at the appendiceal orifice, which may have contributed to incomplete resection [34, 35].

An important consideration when assessing recurrence is accurately identifying the location of previous polypectomy. Although the reliability of careful optical assessment of post-polypectomy scar has been demonstrated and routine biopsies are not necessary, small polypectomy scars can be difficult to confidently locate [36, 37]. Literature comparing frequency of scar identification post-cold polypectomy for smaller polyps is sparse. Like Mass et al., we found that scar identification and documentation is infrequent in everyday practice [20]. Scar identification could be aided by uncommonly employed techniques such as tattoo, clips, cautery, or hot snare for smaller lesions; however, all these techniques come at a cost and add more time to the procedure.

Our secondary outcome was to assess additional lesions at surveillance. We found a clinically significant rate of “very-high-risk lesions” on surveillance in an endoscopy unit with high performance on validated quality indicators. The prevalence of very-high-risk polyps at SC1 was 15%. Overall, those at highest risk were more likely to be older and have a larger polyp resected. Although age and polyp size have been previously shown to be a potential predictors of the risk of additional polyps, it is disappointing that the prevalence of very-high-risk polyps should be so high 6 months after a high-quality colonoscopy [38].

While the strength of this study is the real-world nature of the outcomes, the heterogeneity in practice from multiple operators and retrospective nature of the study is a limitation. The decision to perform cold versus hot snare resection was guided by individual clinician. Larger and more advanced conventional adenomas were more likely to be treated with hot snare resection technique compared with smaller and/or serrated lesions. Hence comparison of recurrence risk according to resection technique is confounded by selection bias and should be interpreted with caution. Similarly, the interval for returning for surveillance was also guided by the proceduralist which introduces selection bias. Furthermore, the cohort size limits evaluation of subgroups that might benefit from lengthening surveillance interval and there may have been differences that the study did not detect.

Although serrated lesions have a low risk of recurrence, the finding of high-risk additional polyps is not negligible and should be an important consideration. The recurrence rates for conventional adenomas were reasonably high and not dependent on size. For hot snare piecemeal polypectomy, this is likely to change as margin thermal ablation post-polypectomy becomes the universal standard of care. Whether there is a role for ablation post-cold snare polypectomy as a simple auxiliary technique to eradicate the possibility of microscopic polyp remnants despite overlapping snare and side resection remains an unexplored concept.

This real-world study shows that cold piecemeal polypectomy even for smaller lesions is a significant contributor to the burden of surveillance. Most of the cases were performed by cold snare technique which enhances safety but limits adoption of adjuvant techniques to lower recurrence and limits chances of identifying previous polypectomy location. In our study, piecemeal polypectomy was associated with a clinically significant risk of recurrence in conventional adenomas even < 20 mm. Especially while the optimal approach for medium-sized (10–19 mm) polyps is still debated, our findings support the current conservative approach with the recommendation of first surveillance at 6 months to ensure early detection and management. Our findings suggest a potential difference between recurrence risk based on histology. However, given the retrospective design introducing bias and risk of confounders, these results are more likely to be hypothesis generating and require validation. Acknowledging the low event rate and limitations in the robustness of multivariate modeling, we hypothesize that if delayed surveillance was to be considered, younger patients with index serrated lesions may be the most suitable candidates to be considered. This suggestion requires larger prospective studies to confirm.

## Supplementary Information

Below is the link to the electronic supplementary material.

Supplementary file1 (DOCX 128 KB)

## Data Availability

The Royal Brisbane and Women’s Hospital (RBWH) and Surgical Treatment and Rehabilitation Service (STARS) maintain a prospective database (Colonoscopy Outcomes Registry) of every patient who has undergone colonoscopy from February 2018 onwards. Post-procedure and subsequent availability of histology results, surveillance nurses dedicated to this task manually enter patient demographics and procedure details, including bowel preparation quality, completion of procedure as well as polyp numbers, size, and location. Lastly, the histology findings, procedure details, and subsequent surveillance recommendations are all entered in a standardized format on a secure web application (REDCap).
