# Supplementary material for: Surveillance Outcomes in an Australian Cohort Undergoing Piecemeal Polypectomy for Conventional Adenomas and Serrated Lesions
Source: Dig Dis Sci. 2026 Jun 4;71(10):4842–53. doi: 10.1007/s10620-026-10024-6 (PMC13558330; doi:10.1007/s10620-026-10024-6)
Supplement: Supplementary file 1 [file 10620_2026_10024_MOESM1_ESM.docx]

**Supplementary Table 1.** Definitions of ‘high-risk’ and ‘very high-risk’ polyps as per the Australian surveillance guidelines.

|  | **‘High-risk’** | **‘Very high-risk’** |
| --- | --- | --- |
| Conventional adenomas | - 1-2 tubular adenomas with HGD or tubulovillous or villous adenomas (with or without HGD), where the size of one or both is ≥10mm or - 3-4 tubular adenomas, where the size of one or more is ≥10mm or - 3-4 tubulovillous and/or villous adenomas and/or HGD, all <10mm or - 5-9 tubular adenomas <10mm without HGD | - 5–9 adenomas where the size of at least one is ≥10 mm or with HGD or villosity or - ≥10 adenomas or - Any polyp requiring piecemeal polypectomy |
| Serrated lesions* | - 3–4 in total sessile serrated lesions, all <10mm without dysplasia or - 1–2 in total sessile serrated lesions ≥10mm or with dysplasia, or hyperplastic polyp ≥10mm or - 1–2 in total traditional serrated lesions, any size. | - ≥5 sessile serrated lesions <10mm without dysplasia or - 3-4 sessile serrated lesion with one or more >10mm or with dysplasia or - 3-4 in total with at least one traditional serrated adenoma any size or - Any lesion requiring piecemeal polypectomy |
| Low risk conventional adenomas <10mm without HGD or villosity  and synchronous serrated lesions | - 3–9 in total, all sessile serrated lesions < 10mm without dysplasia or - 2–4 in total, any serrated lesion ≥10mm and/or dysplasia or - 2–4 in total, any traditional serrated adenoma | - ≥10 in total, all sessile serrated adenomas <10mm without dysplasia or - ≥5 in total, any serrated polyp ≥10mm and/or dysplasia or - ≥5 in total, any traditional serrated adenoma or - Any lesion requiring piecemeal polypectomy |
| High-risk conventional adenoma (tubulovillous/villous adenoma +/- HGD and +/- size ≥10mm) & synchronous lesions | - 2 in total, sessile serrated lesion or traditional serrated adenoma | - ≥3 in total polyps or - Any lesion requiring piecemeal polypectomy |

*Excluding hyperplastic polyps <10mm

**Supplementary table 2** Characteristics conventional adenomas and outcomes of resection in 119 patients with 130 polyps resected with cold and hot snare polypectomy technique.

|  | <20mm | ≥20mm |  |
| --- | --- | --- | --- |
| **Patient characteristics (%)** | **N=50 (42)** | **N=69 (58)** | **p-value** |
| Age, mean (SD), years | 64.4 (11.3) | 66.4 (11.0) | 0.33† |
| Female, n (%) | 18 (36.0) | 31 (44.9) | 0.33 |
| **Polyp characteristics** | N=53 | N=77 |  |
| *Size* |  |  |  |
| median (IQR), mm | 15 (12-15) | 30 (20-40) | <0.001 |
| *Location,* n (%) |  |  |  |
| Right colon | 48 (91) | 51 (66) | **0.001*** |
| Exact location |  |  | **0.003*** |
| Rectum | 0 (0) | 10 (13) |  |
| Sigmoid, descending colon, splenic flexure | 5 (9) | 16 (21) |  |
| Transverse colon, hepatic flexure | 8 (15) | 11 (14) |  |
| Ascending colon | 21 (40) | 28 (36) |  |
| Caecum, IC valve, appendiceal orifice | 19 (36) | 12 (16) |  |
| *Histology* |  |  | **<0.001*** |
| Tubular adenoma with low grade dysplasia (LGD) | 35 (66) | 36 (47) |  |
| Tubular adenoma with high grade dysplasia (HGD) | 5 (9) | 5 (6) |  |
| Tubulovillous adenoma with LGD | 12 (23) | 14 (18) |  |
| Tubulovillous adenoma with HGD | 1 (2) | 22 (29) |  |
| **Polypectomy technique, n (%)** |  |  |  |
| Use of electrocautery for resection | 6 (11) | 42 (55) | **<0.001** |
| Submucosal injection | 36 (68) | 69 (90) | **0.002** |
| Snare tip soft coagulation (STSC) post polypectomy | 2 (4) | 19 (25) | **0.001*** |
| Clip placed | 6 (11) | 20 (26) | **0.040** |
| Site tattooed | 1 (2) | 17 (22) | **<0.001*** |
| **Outcome at SC1** |  |  |  |
| Time from index colonoscopy to first surveillance, median (IQR), months | 7.0 (6.0-12.0) | 6.0 (3.0-6.0) | **<0.001** |
| Time to SC1 <6 months | 10 (19) | 38 (49) | **<0.001** |
| Time to ≥6 months | 43 (81) | 39 (51) |  |
| Confident scar identification | 30 (57) | 60 (78) | **0.010** |
| Suspected macroscopic recurrence | 10 (19) | 11 (14) | 0.49 |
| Biopsy/resection | 16 (30) | 32 (42) | 0.19 |
| Histological recurrence | 8 (15) | 10 (13) | 0.73 |

SD, Standard deviation; N, frequency; Values are n (%) unless otherwise defined; *Fishers exact test; †Two sample t test

**Supplementary table 3.** Characteristics of serrated polyps and outcomes of resection in 83 patients with 91 polyps resected with cold and hot snare polypectomy technique.

|  | <20mm | ≥20mm |  |
| --- | --- | --- | --- |
| **Patient characteristics (%)** | **N= 55 (66.0)** | **N=28 (33.7)** | **p-value** |
| Age, mean (SD), years | 55.7 (14.3) | 58.2 (12.4) | 0.44 |
| Sex, female, n (%) | 40 (72.7) | 20 (71.4) | 0.90 |
| **Polyp characteristics** | **N=61** | **N=30** |  |
| *Size* |  |  |  |
| median (IQR), mm | 14 (10-15) | 21 (20-25) |  |
| *Location, n (%)* |  |  |  |
| Right colon | 51 (84) | 28 (93) | 0.32 |
| Exact location |  |  | 0.06* |
| Rectum | 1 (2) | 1 (3) |  |
| Sigmoid, descending colon, splenic flexure | 9 (15) | 1 (3) |  |
| Transverse colon, hepatic flexure | 14 (23) | 5 (17) |  |
| Ascending colon | 21 (34) | 19 (63) |  |
| Caecum, IC valve, appendiceal orifice | 16 (26) | 4 (13) |  |
| *Histology,* n (%) |  |  | 0.15* |
| Hyperplastic polyp | 8 (13) | 1 (3) |  |
| Sessile serrated lesion | 50 (82) | 25 (83) |  |
| Sessile serrated lesion with dysplasia | 1 (2) | 3 (10) |  |
| Traditional serrated lesion | 2 (3) | 1 (3) |  |
| **Polypectomy technique** |  |  |  |
| Use of electrocautery for resection | 0 | 5 (17) | **0.003*** |
| Submucosal injection | 47 (77) | 24 (80) | 0.75 |
| Snare tip soft coagulation post polypectomy | 1 (2) | 1(3) | 1.00* |
| Clip(s) placed | 5 (8) | 3 (10) | 1.00* |
| Site tattooed | 2 (3) | 1 (3) | 1.00* |
| **Outcome at SC1** |  |  |  |
| Time from index procedure to first surveillance, median (IQR), months | 9.0 (6.0-13.0) | 6.0 (4.0-9.0) | **<0.001** |
| Time to SC1 <6 months | 4 (7) | 10 (33) | **0.002*** |
| Time to SC1 ≥6 months | 57(93) | 20(67) |  |
| Confident scar identification | 18 (30) | 17 (57) | **0.012** |
| Suspected macroscopic recurrence | 4 (7) | 2 (7) | 1.00* |
| Biopsy/resection | 7 (11) | 8 (27) | 0.07 |
| Histological recurrence | 3 (5) | 0 (0) | 0.55* |

SD, Standard deviation; N, frequency; Values are n (%) unless otherwise defined. *Fishers exact test; †Two sample t test

**Supplementary table 4.** Factors associated with additional high or very high-risk polyps at first surveillance colonoscopy among 202 patients with index polypectomy performed with either hot or cold snare polypectomy technique.

|  | No additional or low risk polyps  N=148 (73%)* | High-risk and very high-risk polyps  N=54 (27%)* | p-value |
| --- | --- | --- | --- |
| Age, mean (SD), years | 60.7 (13.0) | 64.8 (12.6) | 0.046* |
| Female, n (%) | 83 (56.1) | 26 (48.1) | 0.34 |
| Polyp histology |  |  |  |
| Serrated | 66 (44.6) | 17 (31.5) | 0.10 |
| Conventional adenoma | 82 (55.4) | 37 (68.5) |  |
| Polyp location |  |  |  |
| Right colon | 115 (77.7) | 47 (87.0) | 0.15 |
| Left colon | 32 (21.6) | 7 (13.0) |  |
| Polyp size (mm), median (IQR) | 18.0 (14.0-25.0) | 20.0 (15.0-30.0) | 0.022† |
| Polyp size |  |  |  |
| <20mm | 83 (56.0) | 22 (40.7) | 0.059 |
| ≥20mm | 65 (44.0) | 32 (59.3) |  |

SD, Standard deviation; N, frequency; Values are n (%) and Pearson’s chi-squared are reported unless otherwise defined; * Two sample t test; †Wilcoxon rank-sum

**Supplementary Table 5.** Second surveillance outcome for 124/181 individuals with conventional adenomas and serrated lesions resected piecemeal using either cold or hot snare polypectomy technique at index colonoscopy.

|  | Conventional adenomas (n=76) | | Serrated lesions (n=48) | |
| --- | --- | --- | --- | --- |
|  | <20mm | ≥20mm | <20mm | ≥20mm |
|  | n=24 | n=52 | n=26 | n=22 |
| Time from SC1 to SC2, median (IQR), months | 14 (8) | 12 (6) | 17 (24) | 15 (16.8) |
| Histological recurrence | 0 | 4 | 1 | 0 |
| Scar identified at SC1 in those with recurrence at SC2 | n/a | 4 | 1 | n/a |
| Scar previously biopsied | n/a | 2 | 0 | n/a |
| High-risk additional lesions (%) | 2 (8) | 2 (4) | 5 (19) | 2 (9) |
| Very high-risk additional lesions (%) | 6 (25) | 8 (15) | 2 (8) | 3 (13) |
